# Atypical Presentation of Infective Endocarditis: Reactive Arthritis as the First Clinical Clue

**DOI:** 10.7759/cureus.100105

**Published:** 2025-12-26

**Authors:** Ahmed H Ahmed, Bayadir A Adam, Samaira K Rafiqui, Aya Dafalla, Ashraf Mukhtar, Omar Aljaziri, Alaa Abdelhamid, Yaman Al-Haneedi, Shouq Al-Enazi, Eman Almaraghi

**Affiliations:** 1 Internal Medicine, Hamad Medical Corporation, Doha, QAT; 2 Medical Education, Hamad Medical Corporation, Doha, QAT; 3 Internal Medicine, Taylor's University, Subang Jaya, MYS; 4 Radiology, Hamad Medical Corporation, Doha, QAT; 5 College of Medicine, Qatar University, Doha, QAT

**Keywords:** angiovac extraction, atypical presentation, infective endocarditis, leukocytoclastic vasculitis, mitral valve vegetation, reactive arthritis, staphylococcus aureus bacteremia

## Abstract

The clinical manifestations of infective endocarditis (IE) may be diverse and atypical, frequently mimicking rheumatologic or autoimmune disorders. The diagnosis of *Staphylococcus aureus* bacteremia may be delayed if early symptoms are nonspecific, despite the significant risk of underlying IE. We report a 29-year-old previously healthy male who presented with a sudden onset of fever, polyarthralgia, bilateral knee effusions, and a widespread erythematous petechial rash. Leukocytosis, thrombocytopenia, transaminitis, and significantly increased inflammatory markers were found during his initial assessment. Blood cultures revealed methicillin-sensitive *Staphylococcus aureus*, despite transthoracic echocardiography showing no vegetations. IE was later confirmed by transesophageal echocardiography. Following successful percutaneous AngioVac-assisted vegetation extraction (AngioDynamics, Inc., Latham, NY), the patient experienced complete recovery, and heart function returned to baseline. He completed a six-week course of intravenous cefazolin. This case emphasizes the challenges in diagnosing atypical presentations of IE, such as reactive arthritis and leukocytoclastic vasculitis. It highlights the need for early transesophageal imaging and the limits of transthoracic echocardiography. Additionally, it demonstrates the success of AngioVac extraction for a left-sided vegetation in select patients. Consideration of IE should be prompted by atypical manifestations such as rash and sterile inflammatory arthritis, especially when *S. aureus* bacteremia is present.

## Introduction

Infective endocarditis (IE) is a serious and potentially fatal condition caused by microbial infection of the endocardial surface of the heart, most commonly involving the cardiac valves [1]. Despite advances in diagnostic modalities and antimicrobial therapy, IE continues to pose significant clinical challenges due to its variable and often nonspecific presentation. The 2023 Duke-International Society for Cardiovascular Diseases (ISCVID) criteria remain widely utilized for diagnosis; however, their accuracy is not absolute, underscoring the importance of maintaining a high index of suspicion, particularly in patients with predisposing conditions such as rheumatic heart disease, prosthetic valves, or a history of intravenous drug use [2].

IE can manifest with a broad spectrum of clinical features, some of which overlap with autoimmune or rheumatologic diseases, contributing to potential delays in diagnosis or misclassification [3]. Musculoskeletal symptoms represent one of the less recognized but clinically important manifestations. Across nine studies, up to 44% of patients with IE reported musculoskeletal complaints, most commonly arthralgia, myalgia, or lower back pain [4].

Reactive arthritis (ReA) is an aseptic inflammatory arthritis that arises following an extra-articular bacterial infection, most often of the genitourinary or gastrointestinal tract [5]. The condition is characterized by sterile synovial fluid and is thought to result from immune complex deposition or molecular mimicry triggered by systemic infection [6]. Post-infectious, immune-mediated reactive arthritis associated with atypical pathogens, including staphylococcal species from bacteremia or IE, is uncommon [7].

Here, we describe a rare case of *Staphylococcus aureus* IE complicated by reactive arthritis, emphasizing the diagnostic challenges and the importance of correlating infectious, cardiac, and rheumatologic findings to ensure timely recognition and effective management.

## Case presentation

We present a case of a 29-year-old previously healthy male who arrived at the emergency department with a three-day history of fever, polyarthralgia involving the right elbow, left shoulder, and both knees, and a progressive generalized erythematous rash. He denied sore throat, nasal congestion, cough, gastrointestinal symptoms, recent travel, sick contacts, or previous similar episodes. There were no features suggesting autoimmune disease, and his past medical history was notable only for intravenous hormonal drug use for bodybuilding in 2022, with no recent dental procedures.

**Figure 1:**
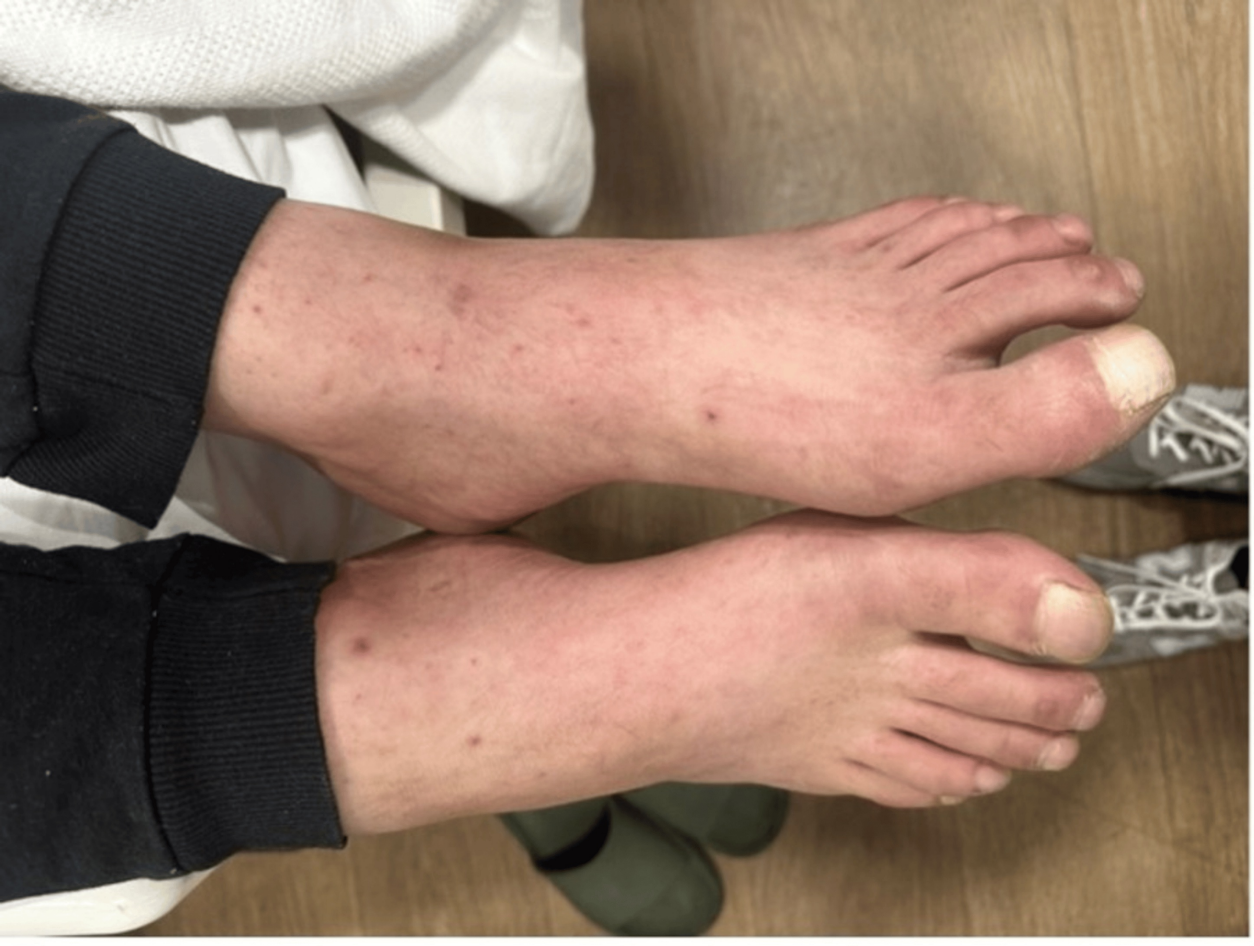
Petechial erythematous rash at initial presentation.

**Figure 2:**
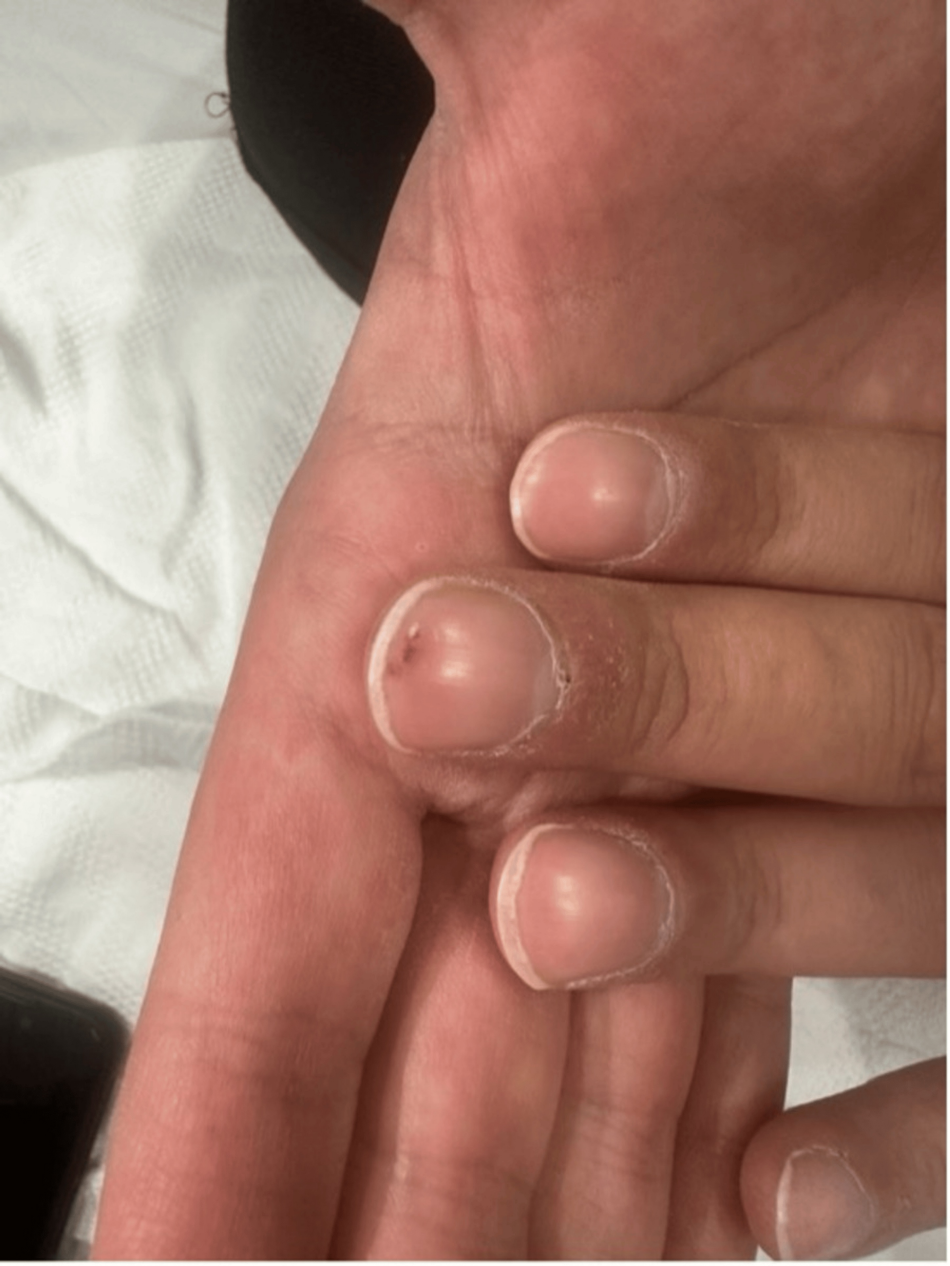
Tender nodule on the nail bed of the left middle finger.

**Figure 3:**
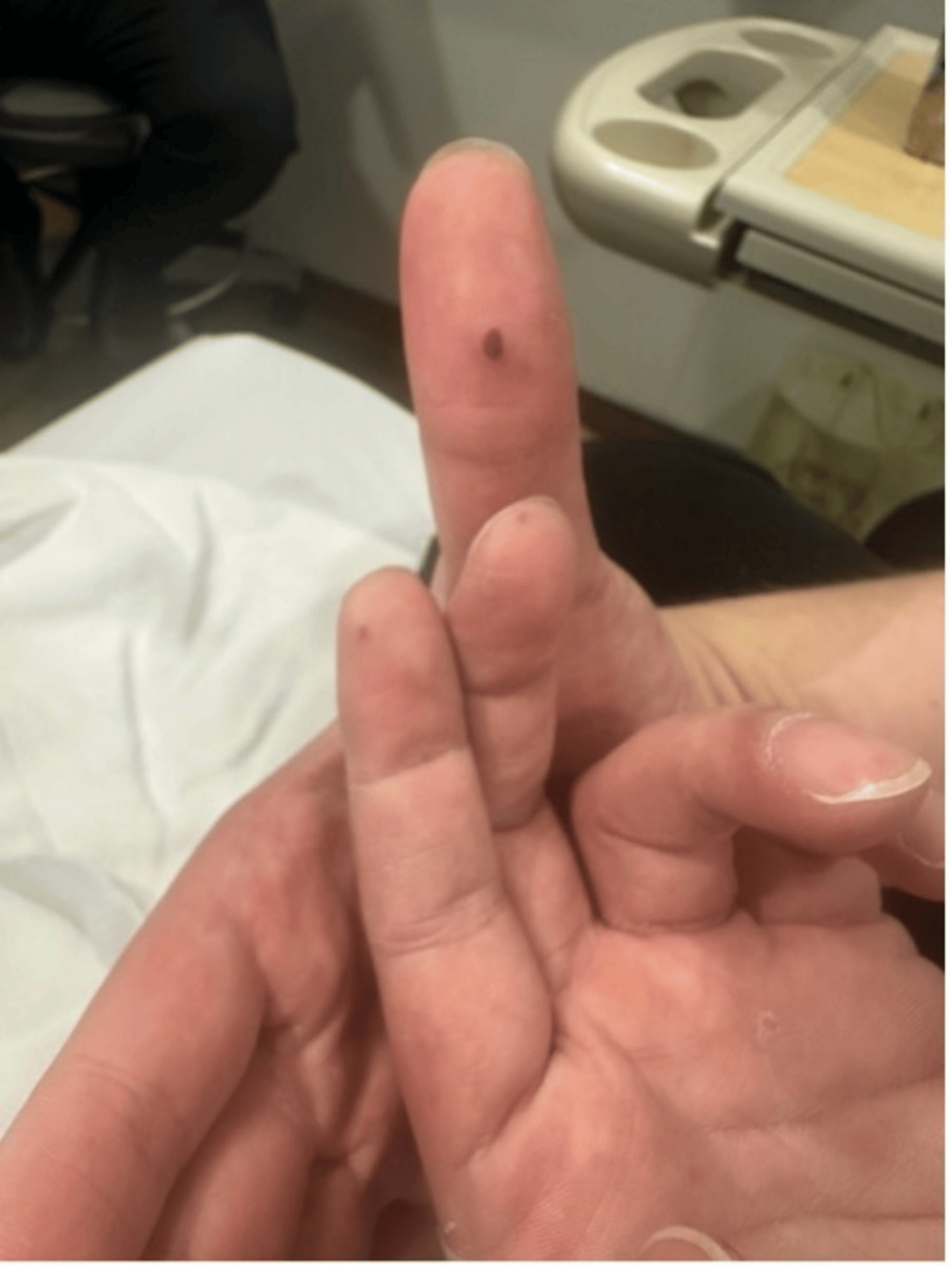
Tender nodule over the thumb.

On arrival, he was febrile (38.3°C), tachycardic (119 bpm), and normotensive. Examination images are shown in Figures 1-3. Although he reported arthralgia, there was no initial joint swelling. Importantly, the cardiovascular examination was completely normal, with no audible murmurs, and the rest of the cardiopulmonary and abdominal examinations were unremarkable.

Initial laboratory investigations demonstrated leukocytosis, thrombocytopenia, coagulopathy, and markedly elevated inflammatory markers (Table 1). Given the combination of fever, rash, and polyarthralgia, the differential diagnosis included IE, systemic vasculitis, and viral myocarditis. Blood cultures were obtained, and empirical intravenous ceftriaxone and vancomycin were initiated. A transthoracic echocardiogram (TTE) performed on admission showed no valvular vegetations but revealed moderate global left ventricular hypokinesis with an ejection fraction of 39%. Cardiology was then consulted, and treatment with valsartan, bisoprolol, and dapagliflozin was started for presumed sepsis-related cardiomyopathy.

**Table 1:** Initial laboratory findings.

Parameter	Result	Units	Reference range
White blood cells	11.4	×10⁹/L	4.0–10.0
Red blood cells	5.4	×10¹²/L	4.5–5.9
Hemoglobin	16.6	g/dL	13.5–17.5
Platelets	103	×10⁹/L	150–400
Prothrombin time	15.6	s	11–14
International normalized ratio	1.4	—	0.8–1.2
C-reactive protein	310	mg/L	<5

Dermatological evaluation revealed generalized erythematous plaques with petechial elements, consistent with leukocytoclastic vasculitis secondary to infection, for which topical mometasone was prescribed. A few days later, methicillin-sensitive *Staphylococcus aureus* (MSSA) was isolated in two sets of blood cultures, and antibiotics were de-escalated to intravenous cefazolin. Microbiological findings are summarized in Table 2.

**Table 2:** Microbiology.

Date collected	Specimen	Result	Sensitivity
11/09	Blood culture	Staphylococcus aureus	Cloxacillin, cefazolin, clindamycin
12/09	Blood culture	Staphylococcus aureus	Same sensitivity
14/09	Synovial fluid	No growth	—
17/09	Mitral valve tissue	Staphylococcus aureus	—

Despite pathogen-directed therapy, the patient’s joint pain progressed, and he developed bilateral knee effusions. Ultrasound-guided aspiration of the right knee joint yielded 2 mL of turbid yellow synovial fluid. Fluid analysis results are shown in Table 3, supporting a diagnosis of reactive arthritis secondary to *S. aureus* bacteremia rather than septic arthritis. Intra-articular corticosteroid therapy was administered as advised by rheumatology.

**Table 3:** Synovial fluid analysis.

Parameter	Result	Units	Reference range
Appearance	Turbid	—	Clear
Color	Yellow	—	Straw
WBC count	18,125	cells/µL	<200 (normal); >50,000 (septic)
Neutrophils	80	%	—
Culture	No growth	—	—

**Figure 4:**
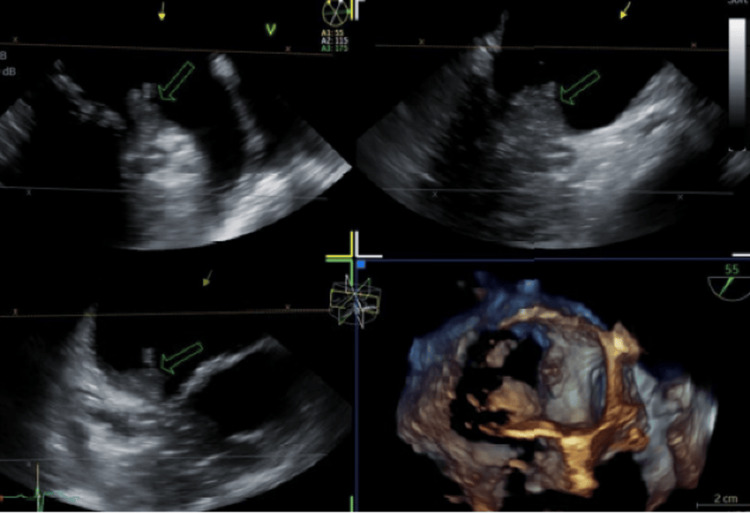
Mobile vegetation on the posterior mitral leaflet’s atrial surface (green arrow).

**Figure 5:**
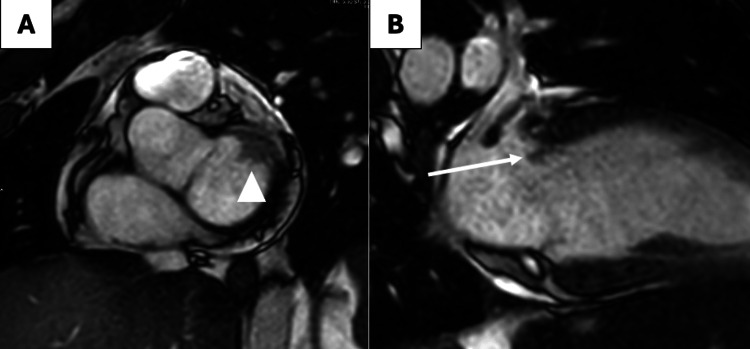
Cardiac MRI in short axis (A) and two-chamber (B) planes showing soft tissue structure attached to the mitral annulus at the atrial side (arrowhead) with a small mobile floating portion (arrow).

The evolving clinical picture, positive blood cultures, and initial TTE findings prompted a transesophageal echocardiogram (TEE), which demonstrated a 1 mm mobile vegetation on the atrial surface of the posterior mitral leaflet with a suspected small abscess cavity (Figure 4), confirming mitral valve infective endocarditis. To assess myocardial involvement, a cardiac MRI was performed (Figure 5), demonstrating no myocardial edema or fibrosis, thereby ruling out myocarditis and supporting septic cardiomyopathy as the cause of transient left ventricular (LV) dysfunction.

Given the presence of mobile vegetation and the associated embolic risk, the cardiology team selected percutaneous AngioVac-assisted extraction (AngioDynamics, Inc., Latham, NY) as a minimally invasive alternative to surgery. The TEE-guided AngioVac aspiration achieved complete removal of the vegetation, and post-procedural imaging confirmed resolution. Histopathological examination of the extracted material revealed fibrin, inflammatory exudate, and Gram-positive cocci in clusters consistent with *Staphylococcus aureus*. A small fibrinous thrombus was also retrieved, supporting septic embolization.

Following the extraction, the patient’s condition improved rapidly. Within 48 hours, his rash and inflammatory markers began to decline, and a repeat echocardiogram showed normalized left ventricular ejection function (LVEF = 55%). Persistent knee pain responded well to intra-articular methylprednisolone, with complete resolution of joint swelling and restoration of mobility.

**Figure 6:**
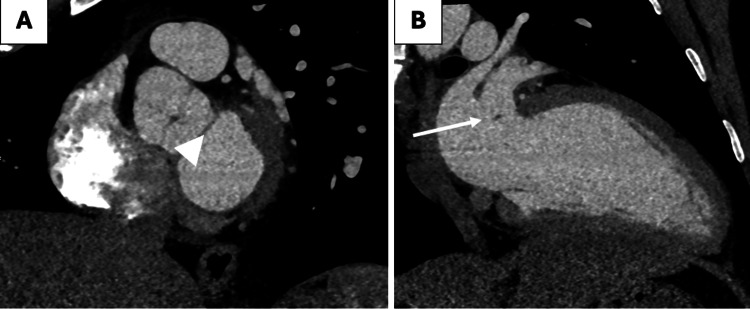
Cardiac CT in short axis (A) and two-chamber (B) planes post intervention showing a significant reduction in size of both the sessile (arrowhead) and mobile (arrow) post-catheter intervention with minimal residue.

A follow-up cardiac CT scan demonstrated a small (2 mm) tissue irregularity at the mitral annulus consistent with post-procedural changes and without residual abscess or vegetation (Figure 6). He completed a full six-week course of intravenous cefazolin, and a follow-up TEE and PET-CT are planned to confirm eradication of infection and assess long-term cardiac recovery.

## Discussion

IE caused by *Staphylococcus aureus* is associated with substantial morbidity and mortality and requires a high index of suspicion, particularly when clinical features are atypical. Among patients with *S. aureus* bacteremia (SAB), IE may be clinically occult at presentation, and reliance on TTE alone may lead to missed diagnoses, as its sensitivity for detecting vegetations is significantly lower compared to TEE [8]. Current risk-stratification tools, such as the VIRSTA score and the POSITIVE and PREDICT models, highlight that up to one-third of SAB cases may harbor underlying IE, underscoring the need for early definitive imaging in patients with systemic manifestations or bacteremia persistence [9,10]. In our patient, the absence of cardiac murmurs and an initially unremarkable TTE did not exclude IE, and the subsequent TEE played a crucial role in establishing the diagnosis by identifying a small mobile vegetation with a suspected abscess cavity.

Musculoskeletal and rheumatologic manifestations are well-documented but often underrecognized complications of IE. Arthralgia, myalgia, and back pain have been reported in up to 44% of cases, frequently preceding definitive cardiac findings and occasionally mimicking primary rheumatic disease [11]. Reactive arthritis secondary to *S. aureus* infection, although rare, has been described in the literature, typically presenting with sterile synovial fluid and occurring in the setting of systemic bacteremia or endocarditis [12]. In our case, the patient developed worsening polyarthralgia with knee effusions despite pathogen-directed therapy. Synovial fluid analysis revealed a markedly elevated neutrophilic count with negative cultures, consistent with reactive arthritis rather than septic arthritis, supporting the immune-mediated pathogenesis described in prior reports.

The patient also exhibited a generalized erythematous and petechial rash, which dermatology identified as leukocytoclastic vasculitis. Cutaneous small vessel vasculitis is recognized as a potential extra-cardiac manifestation of IE, occurring through immune complex deposition during active infection [13]. Similar presentations have been described in cases of endocarditis-associated IgA vasculitis, underscoring the diverse immunologic responses triggered by cardiac infections [14]. In this context, the coexistence of reactive arthritis and leukocytoclastic vasculitis further illustrates the complex interplay between infection and host immune response.

Cardiac MRI was pursued due to the initial reduction in left ventricular ejection fraction and concern for myocarditis. MRI has emerged as a valuable tool for distinguishing septic cardiomyopathy from true infectious myocarditis or myocardial abscess formation, and its utility in our patient supported the diagnosis of reversible septic cardiomyopathy [15].

Management of IE in this case was further complicated by the presence of a mobile vegetation with embolic potential. Surgical intervention is typically indicated for large vegetations, persistent bacteremia, or evidence of systemic embolization. However, for selected patients, percutaneous vacuum-assisted aspiration using the AngioVac system has emerged as a minimally invasive alternative, particularly for right-sided lesions or in patients who are poor surgical candidates [16]. Although its use in left-sided vegetations is less common and technically challenging, several case reports have demonstrated successful removal of vegetations and rapid clearance of bacteremia using AngioVac-directed therapy [17,18]. In our patient, the multidisciplinary decision to proceed with AngioVac extraction resulted in the complete removal of the mitral vegetation and was followed by prompt clinical improvement.

This case underscores several important clinical lessons. First, IE must remain a diagnostic consideration in patients presenting with fever, rash, and polyarthralgia, even when cardiac examination is unremarkable, and initial TTE findings are negative. Second, the co-occurrence of leukocytoclastic vasculitis and reactive arthritis may serve as early immunologic clues to underlying endovascular infection. Third, advanced cardiac imaging, including TEE and MRI, is essential for characterizing structural complications and guiding management decisions. Finally, minimally invasive AngioVac-assisted extraction may be a viable therapeutic option in selected patients with mobile vegetations at high risk for embolization.

## Conclusions

This case highlights the diagnostic complexity of *Staphylococcus aureus* IE, particularly when early manifestations mimic autoimmune or rheumatologic disease. The coexistence of reactive arthritis and leukocytoclastic vasculitis served as important immunologic clues that prompted further investigation despite a normal cardiovascular examination and an initially negative TTE. However, this is a single-case report, and the diagnosis of leukocytoclastic vasculitis was clinical without skin biopsy confirmation, which limits the generalizability of our findings. Early use of TEE, targeted antimicrobial therapy, and a multidisciplinary approach were critical to establishing the diagnosis of IE and guiding management. The successful application of AngioVac-assisted extraction in this left-sided vegetation demonstrates the potential role of minimally invasive techniques in selected patients at high embolic risk. Ultimately, timely recognition and comprehensive evaluation led to full clinical recovery, emphasizing the need for heightened clinical vigilance when confronted with atypical presentations of IE.
